# How to elicit a negative bias? Manipulating contrast and saturation with the facial emotion salience task

**DOI:** 10.3389/fpsyg.2024.1284595

**Published:** 2024-08-29

**Authors:** Sarah Tholl, Christian A. Sojer, Stephanie N. L. Schmidt, Daniela Mier

**Affiliations:** Department of Psychology, University of Konstanz, Konstanz, Germany

**Keywords:** salience, facial affect recognition, schizophrenia, schizotypy, negative bias

## Abstract

**Introduction:**

Emotion recognition impairments and a tendency to misclassify neutral faces as negative are common in schizophrenia. A possible explanation for these deficits is aberrant salience attribution. To explore the possibility of salience driven emotion recognition deficits, we implemented a novel facial emotion salience task (FEST).

**Methods:**

Sixty-six healthy participants with variations in psychometric schizotypy completed the FEST. In the FEST, we manipulated physical salience (FEST-1: contrast, FEST-2: saturation) of emotionally salient (positive, i.e., happy and negative, i.e., fearful) and non-salient (neutral) facial expressions.

**Results:**

When salience was high (increased contrast), participants recognized negative facial expressions faster, whereas neutral faces were recognized more slowly and were more frequently misclassified as negative. When salience was low (decreased saturation), positive expressions were recognized more slowly. These measures were not associated with schizotypy in our sample.

**Discussion:**

Our findings show that the match between physical and emotional salience influences emotion recognition and suggest that the FEST is suitable to simulate aberrant salience processing during emotion recognition in healthy participants.

## 1 Introduction

The recognition of facial expressions allows us to understand others and can be seen as the basis for more complex social-cognitive processes, as well as successful social functioning. In mental disorders such as schizophrenia (SZ), this social-cognitive ability is often impaired (Kohler et al., 2003; Kohler et al., 2010; Savla et al., 2013; Lee et al., 2015) and may lead to misperception of others and reduced social functioning (Hooker and Park, 2002; Aguirre et al., 2008). Specifically the processing of neutral faces seems to be affected (Kohler et al., 2003; Aguirre et al., 2008; Brown and Cohen, 2010; Mier et al., 2014; Derntl and Habel, 2017) with patients showing a negative bias, i.e., a perception of negative emotions in neutral facial expressions (Kohler et al., 2003; Mier et al., 2014). While the cause for this negative bias is still unknown, one possible explanation that arises from neuroimaging findings (Schmidt et al., 2019; Kozhuharova et al., 2020) is the attribution of emotional salience to non-emotional expressions. For the present study, we designed a novel emotion recognition task, manipulating physical and emotional salience with the aim to simulate emotion recognition impairments that occur in schizophrenia in a non-clinical participant sample with varying degrees of schizotypy.

Salience is defined as the distinctiveness of a stimulus (Itti and Koch, 2000). Two major pathways can determine the salience of a stimulus: physical salience and motivational salience. While physical salience is determined by low-level features of a stimulus such as orientation, contrast, or colour, motivational salience depends on the content-related salience of a stimulus, such as a famous person, or an angry facial expression. Emotional facial expressions are inherently motivationally/emotionally salient,^1^ as their recognition is evolutionarily adaptive and highly relevant for communication and survival (Öhman, 2016), leading to more allocation of automatic attention to emotional compared to neutral stimuli (Carretie, 2014). Particularly threatening (fearful and angry) faces are detected automatically and rapidly (Palermo and Rhodes, 2007). Thus, both a physically distinct stimulus and an emotional facial expression can be deemed salient, while a physically unobtrusive stimulus (i.e., a stimulus that is not distinct from other stimuli in terms of its low-level features) and a neutral facial expression seem to be non-salient.

Salience of emotional faces is classically investigated using visual search paradigms such as a “face-in-the-crowd” paradigm, in which an emotional face “pops out” in an array of distractor faces (e.g., Calvo and Nummenmaa, 2008). In comparison to the oddball paradigm, which is also frequently used to investigate stimulus salience (Schlüter and Bermeitinger, 2017), the face-in-the-crowd paradigm presents distractor and target stimuli simultaneously and not consecutively. However, differences in physical salience of targets and distractors, number and similarity of distractors may confound the results (Becker and Rheem, 2020). Eye tracking studies with attention heatmaps have identified salient facial features (such as a smiling mouth) that seem to guide visual search (e.g., Calvo and Nummenmaa, 2008). This is in line with the idea that generally, facial emotion processing relies on feature-based rather configural mechanisms, with the eyes and mouth as particularly salient visual features due to differences in low-level visual properties (Elsherif et al., 2017). Hence, physically salient features and emotional salience typically correspond, but this congruence may be disturbed in aberrant salience perception.

Aberrant salience perception refers to a perceived distinctiveness of a stimulus that is neither physically nor emotionally salient. The aberrant salience hypothesis of psychosis describes aberrant salience attribution as a mechanism of delusion formation (Kapur, 2003; Kapur et al., 2005): Due to chaotic dopaminergic signalling in the ventral striatum, emotional salience is attributed to non-salient, neutral stimuli. To explain this aberrant salience perception, meaning is then ascribed to the stimulus; i.e. patients find a cognitive explanation of their altered experience and direct further attention to this type of stimuli, e.g., to white cars, or people wearing headphones. By the initial importance of these stimuli, they become more salient and attract more attention, resulting in a self-amplifying process that also influences the emotional response to these stimuli. Thus, aberrant salience perception is assumed as a trigger of cognitive and emotional processes that eventually result in schizophrenia pathology and in particular delusions. There is extensive literature on aberrant salience processing in SZ and psychosis (Menon et al., 2005; Heinz and Schlagenhauf, 2010; Ballerini et al., 2022) with neuroimaging studies showing stronger striatal activation for neutral stimuli in SZ compared to controls in incentive delay and aversive learning tasks (Heinz and Schlagenhauf, 2010; Jensen et al., 2008). To our knowledge however, only one study investigated facial emotional recognition and aberrant salience in acute psychosis, finding a positive association of aberrant salience with the attribution of negative valence to positive and neutral facial expressions (Comparelli et al., 2020).

In addition, functional magnetic resonance imaging studies (fMRI) give indirect evidence for a link of aberrant salience and emotion recognition deficits in SZ, psychosis risk and schizotypy. Psychosis risk was consistently associated with hyperactivity in temporal and frontal regions during neutral face processing (Kozhuharova et al., 2020; Yan et al., 2020). Further, Schmidt et al. (2019) found higher ventral striatum activation for fearful than happy facial expressions during final decisions in a social decision-making task. This effect was pronounced for participants with hasty decision-making, i.e., a jumping to conclusion bias that has also been associated with SZ (Dudley et al., 2016). Esslinger et al. (2012) showed a correlation between nucleus accumbens activation for implicit face perception of salient persons (famousness and high saturation) and reward anticipation in schizophrenia, but no effect of the salience manipulation *per se* on task performance or brain activation in SZ. Amygdala activation has been suggested to signal salience of faces regardless of their emotional salience in healthy participants (Santos et al., 2011) but a specific association of a negative bias with increased amygdala activation to neutral stimuli was found in SZ patients compared to controls, suggesting aberrant salience attribution (Mier et al., 2014).

Taken together, there is at least some evidence that aberrant salience processing is related to emotion recognition deficits and in particular a negative bias. However, to our knowledge, no studies have tried to experimentally induce aberrant salience attribution during emotion recognition. We propose that an increase in the physical salience of emotional facial expressions indicates their greater significance, leading to a congruent effect of physical and emotional salience, and with this improved recognition performance. For neutral stimuli however, an incongruence of high physical salience and lack of emotional salience may simulate aberrant salience processing (hypersalience), and with this a negative bias, i.e., the misperception of emotional salience.

We designed the Facial Emotion Salience Task (FEST) in which we experimentally manipulated both the physical and emotional salience of facial expressions. Positive (happy), negative (fearful) and neutral facial expressions were presented. Physical salience was varied between stimuli in the course of the experiment by increasing or decreasing low-level features of the stimuli (FEST-1: contrast or FEST-2: saturation) compared to the original unmanipulated stimuli. We hypothesized that when physical salience is high compared to unmanipulated, (a) the recognition of emotional faces would be faster and more accurate, whereas (b) the recognition of neutral faces would be slower and less accurate, that is neutral faces would more frequently be miscategorized as emotional (positive or negative). Conversely, when physical salience is low, (c) recognition of neutral faces should be faster and more accurate while (d) emotional face recognition should be slower and less accurate. We had no hypothesis on which physical salience version would lead to more bias. Since emotion recognition deficits are also found in people with an at-risk mental state (Amminger et al., 2012; Allott et al., 2015; Martin et al., 2020) and, although less consistently, in schizotypy (Dickey et al., 2011; Giakoumaki, 2016; Dawes et al., 2021), we also assessed psychometric schizotypy of our healthy participants using the Oxford-Liverpool Inventory of Feelings and Experiences (O-LIFE; Mason et al., 1995; Grant et al., 2013). Schizotypy refers to a set of personality traits that are similar to attenuated SZ symptoms and correlate with an increased psychosis risk (Grant et al., 2018). A negative bias in emotion recognition has been related to disorganized and positive schizotypy (Brown and Cohen, 2010; Statucka and Walder, 2017). In addition, Schmidt and Roiser (2009) found an association of schizotypy with aberrant implicit salience in a salience attribution task, but not with aberrant explicit, self-reported salience. Further, accounts of aberrant salience experience in a self-report measure have also been linked to high schizotypy (Karoly et al., 2015), especially for the positive dimension (Raballo et al., 2019). On a neural level, aberrant dopaminergic transmission has been demonstrated in (positive) schizotypy (Mohr and Ettinger, 2014). Increased Gamma oscillations and prolonged reaction times indicating hypersalience during the processing of physically salient distractors have also been found in positive schizotypy (Kornmayer et al., 2015). Thus, we expected total schizotypy score, positive and disorganized dimensions to (e) predict negative bias and (f) to be associated with an increased effect of physical salience on emotion recognition.

## 2 Methods

### 2.1 Participants

Based on a power analysis with G*Power (Faul et al., 2009), assuming a small to medium effect size of *f* = 0.15 with a power of 0.95, we aimed at 58 participants. Sixty-seven participants were recruited using *SONA Systems* of the University of Konstanz, social media platforms, flyers and word-of-mouth recommendation to allow a dropout rate of 10%. However, only one person had to be excluded due to technical difficulties. Thus, the final sample consisted of 66 participants (34 female, 29 male and 3 nonbinary) with an average age of 22.27 years (SD = 2.44) and mean verbal IQ of 104.23 (SD = 7.98). Before inclusion, prospective participants were screened via telephone. Inclusion criteria were age between 18 and 30 years, higher education entrance certification, normal or corrected-to-normal vision, and fluency in German. Exclusion criteria were neurological illness, current treatment for mental disorders, or Covid-risk status. Data on ethnicity was not collected, however, our sample was predominantly Caucasian.

### 2.2 Procedure

The study took place between June and October 2021, in adherence with a strict Covid safety protocol. First, participants received written and oral information on study procedures and aims, had time to ask questions, and gave written informed consent. Then, participants filled in a verbal intelligence measure (Wortschatztest; Schmidt and Metzler, 1992) in paper-pencil form. Next, the tasks were explained in detail and practiced. Afterwards, participants completed five computerized tasks and 10 questionnaires. Compensation was either two course credits or 20 € for their participation, and an additional 3 to 8 € depending on their winnings in the fourth task. The whole session lasted around 2 h.

### 2.3 Experimental design

The study included five experimental tasks: (1) FEST-1: contrast, (2) FEST-2: saturation, (3) an Emotional Visual Scene Recognition Task (modified from Schettino et al., 2012), (4) a Trust Game (see Erkic et al., 2018), and (5) a Beads Task (Huq et al., 1988). The task order was identical for all participants. Results of task three, four and five (see Supplementary materials) will be presented elsewhere, as they are part of a different research question regarding decision processes.

In the FESTs, physical and emotional salience are combined. Stimuli consisting of a series of photographic faces from the NimStim Face Stimulus Set (http://www.macbrain.org/resources.htm; Tottenham et al., 2009, see Supplementary Tables S8, S9) were presented using Presentation (Version 20.2, Neurobehavioral Systems Inc.). We selected 5 men and 5 women of Caucasian appearance who appeared in both the FEST-1 and the FEST-2, each displaying a positive (happy), negative (fearful) and neutral emotional expression.

**Figure 1:**
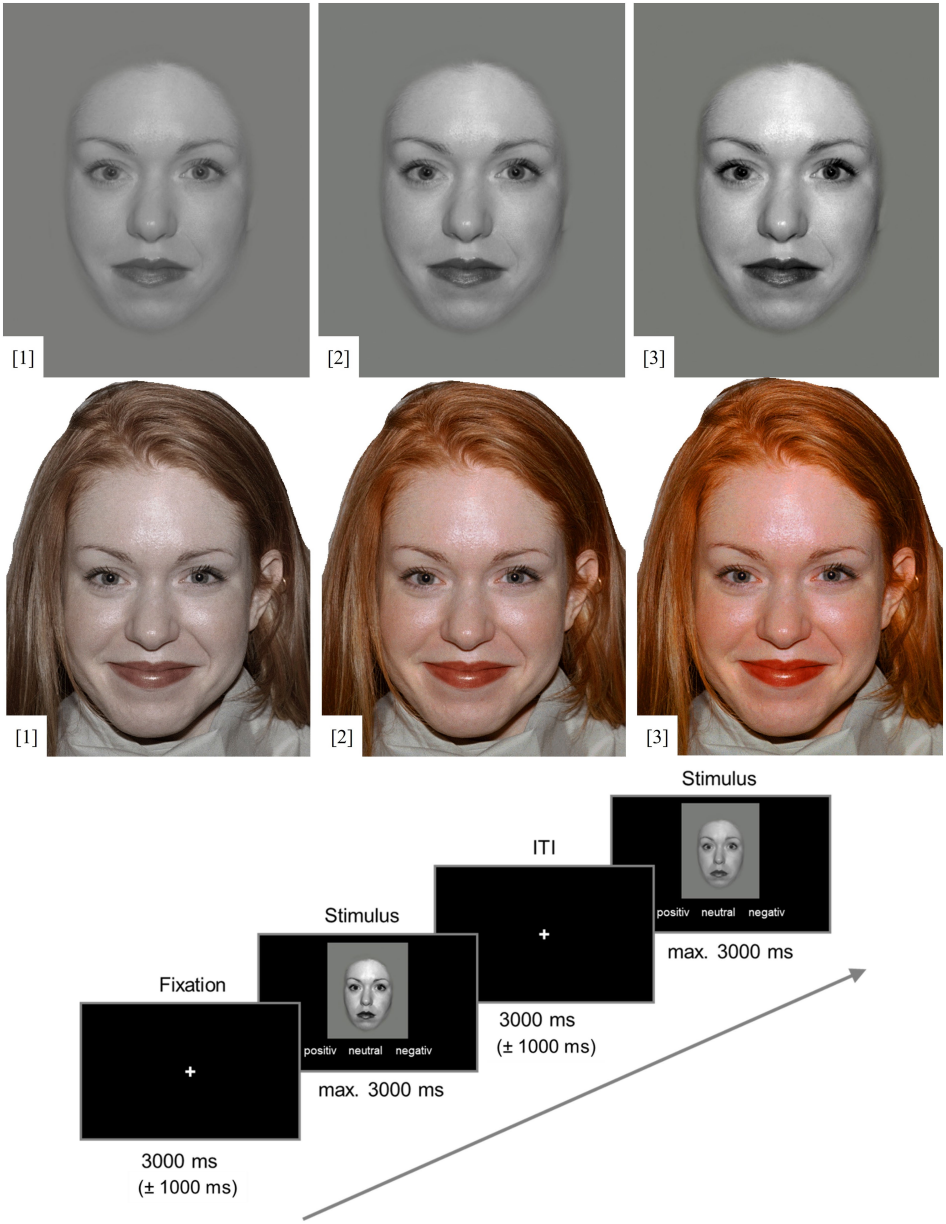
Examples of stimuli and experimental paradigm. Top: contrast condition. Middle: saturation condition. Bottom: experimental paradigm with the example of neutral and negative facial expressions. [1] Low physical salience, [2] unmanipulated, and [3] high physical salience condition; happy facial expression. ITI, inter trial interval. Stimuli were shown until a response was given, but at most for 3,000 ms. Face stimuli were taken from the NimStim data set (Tottenham et al., 2009) which is available to the scientific community at https://danlab.psychology.columbia.edu/content/nimstim-set-facial-expressions. All faces are from the NimStim data set are publicly available for scientific research.

For FEST-1, grayscale pictures of these 5 men and women were adopted from Matzke et al. (2014) with neutral facial expressions, and morphed emotional facial expressions with 40% intensity of happiness or fear. For each facial stimulus, contrast was manipulated using the free GNU Image Manipulation Software (GIMP, Version 2.10.22, 2020). In a high physical salience condition, contrast was increased by 25%. In a low salience condition, contrast was decreased by 25%. In the unmanipulated condition, contrast was unchanged (see Figure 1). This resulted in a total of 9 pictures per stimulus person, and 90 trials overall.

FEST-2 used the original, colour pictures from the NimStim stimulus set. Again, neutral facial expressions, as well as expressions of happiness and fear were shown. In the high physical salience condition, the saturation of each stimulus was increased by 50% using GIMP. In the low physical salience condition, saturation was decreased by 50% (see Figure 1). In the unmanipulated condition, saturation remained unchanged. This, again, resulted in 9 pictures per stimulus person and 90 trials overall.

Stimuli were presented in pseudo-randomized order within each task, ensuring that no stimulus person or emotion were displayed more than twice in a row. The order was identical for all participants. Each face was shown with the response options positive, neutral, and negative below. Participants were instructed to rate the valence of the facial expressions as quickly as possible by pressing the corresponding button on a four-button response pad: left for positive, up for neutral, and right for negative valence. Faces were displayed for up to 3 s. When participants recognized the emotion in less than 3 s, the fixation cross appeared and was added to the time of the jittered inter trial interval (ITI). An example trial with the timing of the experiment is presented in Figure 1. After FEST-1 (greyscale pictures), participants were informed that they would now complete the same task with colourful pictures (FEST-2). The duration of each task was approximately 9 min. All tasks were presented using Presentation (Version 20.2, Neurobehavioral Systems Inc.).

### 2.4 Questionnaires

After the experimental paradigms, participants completed 10 self-report measures using Qualtrics (Version 07/21; Qualtrics, Provo, UT, USA) on a screen in portrait orientation. The two questionnaires that are relevant for the current analyses are the O-LIFE (Mason et al., 1995; Grant et al., 2013) and the Brief Symptom Inventory (BSI; Derogatis, 1978; Franke, 2000).

The O-LIFE (Mason et al., 1995; Grant et al., 2013) captures schizotypy in four dimensions: (1) Unusual Experiences (UnEx) contains items measuring positive schizotypy; (2) Introvertive Anhedonia (IntAn) describes experiences similar to attenuated negative symptoms of schizophrenia; (3) Cognitive Disorganisation (CogDis) contains items describing reduced attention and thought disorder; (4) Impulsive Nonconformity (ImpNon) refers to a lack of self-control. The O-LIFE has 104 items with a dichotomous response format (yes/no). The German version has shown good test–retest-reliability (r = 0.89; Grant et al., 2013). Internal consistency in our sample was good for the UnEx (Cronbach’s α = 0.85) and CogDis (α = 0.83) and adequate for the IntAn (α = 0.65) and ImpNon (α = 0.69) scales.

To investigate possible associations with other psychological symptoms in an exploratory approach, we calculated additional Spearman correlation analyses with the Brief Symptom Inventory (BSI; Derogatis, 1978; Franke, 2000) The BSI captures nine symptom dimensions: Somatization, Obsession-Compulsion, Interpersonal Sensitivity, Depression, Anxiety, Hostility, Phobic Anxiety, Paranoid Ideation, Psychoticism (see Supplementary materials for a description of the BSI and the results of the exploratory analyses).

### 2.5 Analytic strategy

For each stimulus set, 3 (salience) x 3 (valence) repeated measures analyses of variance (ANOVAs) were calculated on the two dependent variables: number of correct responses and reaction times for correct responses. The two task versions were analysed independently. The interaction of the misclassification type and physical salience was explored by calculating 3 (salience) x 6 (misclassification type) repeated measures ANOVAs on the number of misclassifications for each type: neutral as negative, neutral as positive, negative as neutral, negative as positive, positive as neutral and positive as negative. If Mauchly tests revealed violations of sphericity, Greenhouse–Geisser correction was applied. Bonferroni corrections were applied for post-hoc paired *t*-tests and effect sizes (Cohen’s d) were calculated. Internal consistencies for emotion categories were calculated using Cronbach’s alpha.

The association of schizotypy with a negative bias was investigated by regressing the positive, disorganized and total sum scores of the O-LIFE on the number of misclassifications of neutral and positive facial expressions as negative. To assess whether schizotypy modulates the interaction of physical salience and valence in the FEST, difference scores were calculated for each valence, subtracting the number of correct responses as well as correct reaction times for each physical salience condition: high salience minus low salience, high salience minus unmanipulated salience, and unmanipulated salience minus low salience. Using Spearman’s rho correlation with a Bonferroni-Holm correction for multiple testing, association with O-LIFE scores was tested.

### 2.6 Transparency and openness

Logfiles were processed with customized Matlab (R2020b) scripts. All further analyses were performed using IBM SPSS Statistics (Version 26.0; IBM Corp., New York). Detailed information on stimulus material and all analyses that are not reported in the main text can be found in Supplementary Tables S1–S9. The experimental design and its analysis were not pre-registered. Anonymized data and the presentation file can be accessed at https://osf.io/thspd/?view_only=40a7366547314790aca5879c7e84bf4c.

## 3 Results

### 3.1 Descriptives

Participants recognized facial expressions with a mean accuracy of 64.54% (SD = 7.69%) in the FEST-1 (contrast) and 89.70% (SD = 7.69%) in FEST-2 (saturation). Mean reaction times were 991.75 ms (SD = 139.56 ms) for FEST-1 and 849.30 ms (SD = 117.56 ms) in FEST-2. Internal consistency was good for all emotion categories (α ≥ 0.80).

Mean schizotypy sum scores were 27.77 (SD = 12.19, range: 8–64) for the O-LIFE total score, 6.83 (SD = 5.13, range: 0–24) for positive (UnEx) scale, 9.09 (SD = 5.13, range: 0–22) for the disorganized (CogDis) scale, 4.70 (SD = 3.11, range = 0–5) for the negative (IntAn) scale, and 7.15 (SD = 3.50, range: 1–15) for the impulsive nonconformity scale (ImpNon).

### 3.2 Interaction of physical and emotional salience

#### 3.2.1 FEST-1: contrast

**Figure 2:**
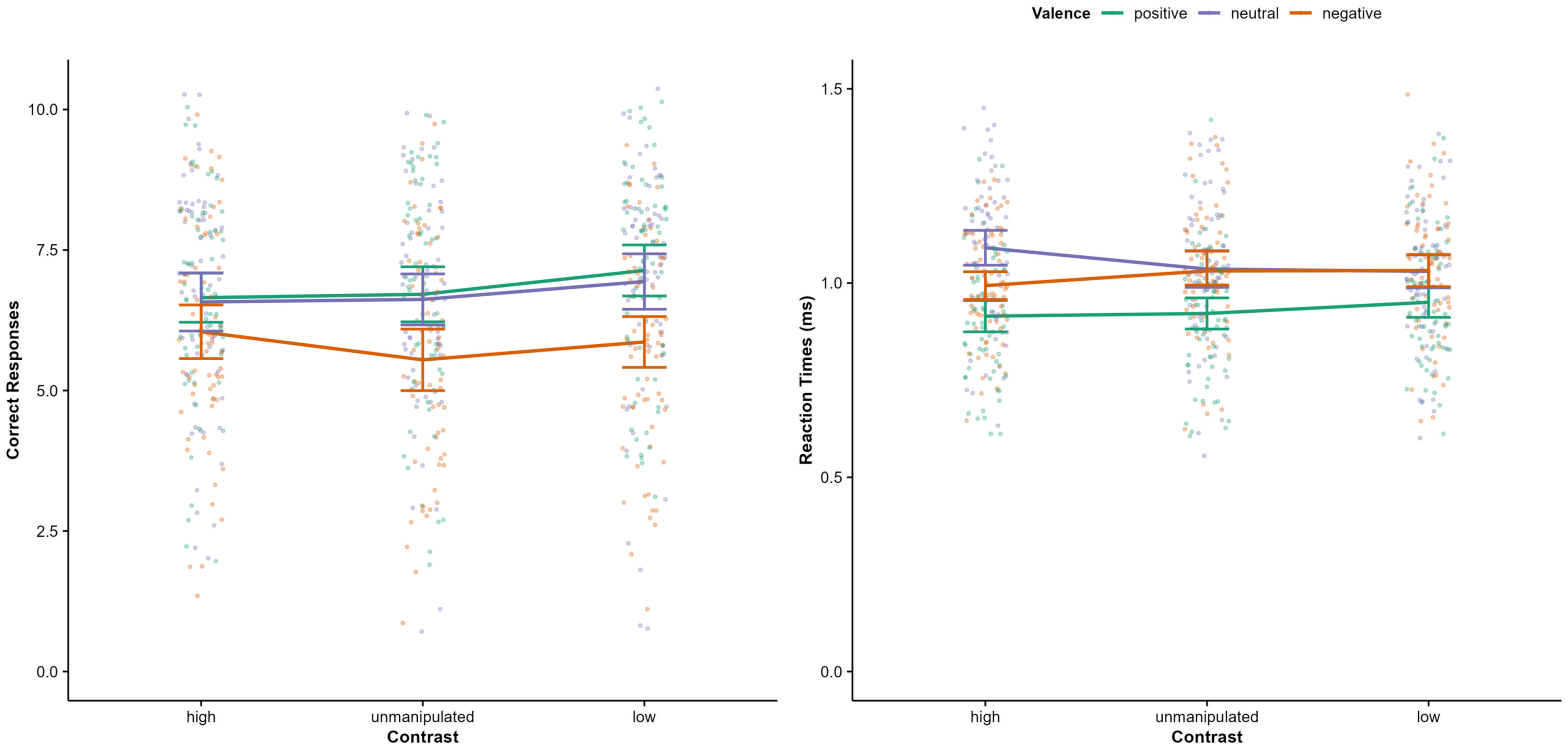
Correct responses and reaction times, separately for valence of facial expression and physical salience condition (contrast). Error bars represent 95% Confidence Intervals.

Significant main effects on accuracy were shown for valence and physical salience (see Table 1). Positive expressions were more often recognized correctly than negative (*p* = 0.005, M_Diff_ = 1.02, 95% CI [0.253, 1.778]), but not neutral expressions (*p* = 1.00). Correct responses for neutral and negative expressions did not differ significantly (*p* = 0.068, d = 0.29). Accuracy was higher in the low physical salience condition than in the unmanipulated condition (*p* = 0.015, M_Diff_ = 0.35, 95% CI [0.054, 0.653]; see Figure 2). The high physical salience condition did not differ significantly from the unmanipulated (*p* = 0.864) and low (*p* = 0.211) physical salience conditions. The physical salience × valence interaction was not significant.

**Table 1:** Within-subjects ANOVA results for FEST-1: contrast.

Effect	*df*	*df(error)*	*F*	*η* ^2^ *_p_*	*p*
Accuracy					
Valence	1.78	115.47	5.65	0.08	0.004
Physical salience	2	130	4.32	0.06	0.015
Valence × physical salience	4	260	2.16	0.03	0.074
Reaction times					
Valence	2	124	38.70	0.38	<0.001
Physical salience	2	124	0.41	0.01	0.666
Valence × physical salience	4	248	8.47	0.12	<0.001
Misclassifications					
Type	2.08	135.21	82.18	0.56	<0.001
Physical salience	2	130	5.59	0.79	0.005
Type × physical salience	7.02	465.58	2.67	0.04	0.01

For reaction times, again, there was a significant main effect for valence (see Table 1): Positive faces were recognized significantly faster than neutral (*p* < 0.001, M_Diff_ = −0.12, 95% CI [−0.159, −0.088]) and negative expressions (*p* < 0.001, M_Diff_ = −0.09, 95% CI [−0.128, −0.058]), which did not differ significantly from each other (*p* = 0.155, M_Diff_ = 0.03, 95% CI [−0.007, 0.068]). Further, there was a significant physical salience x valence interaction. Negative expressions were recognized significantly faster when physical salience was high compared to unmanipulated (*p* = 0.021, M_Diff_ = −0.05, 95% CI [−0.085,-0.005]) and decreased (p = 0.01, M_Diff_ = −0.05, 95% CI [−0.087,-0.009]). Neutral expressions were recognized significantly slower when physical salience was high compared to unmanipulated (*p* = 0.003, M_Diff_ = 0.06, 95% CI [0.017, 0.097]) and low (*p* < 0.001, M_Diff_ = 0.06, 95% CI [0.025, 0.098]; see Figure 2). There were no significant differences for the recognition of positive expressions (all *p*s > 0.155). The main effect for physical salience was not significant.

A main effect for misclassification type was found (see Table 1). The misclassification of negative faces as neutral was significantly more common than all other misclassifications (all *p*s < 0.03). All other misclassification types also significantly differed from each other (positive as negative, positive as neutral, neutral as positive, negative as positive; all *p*s < 0.001, see Supplementary Table S7), except for misclassifications of positive as negative and neutral as positive (*p* = 0.08), as well as neutral as negative and positive as neutral (*p* = 0.53). A significant misclassification type x physical salience interaction was found. As hypothesized, neutral expressions were more often classified as negative when physical salience was high compared to low (*p* = 0.039, M_Diff_ = 0.424, 95% CI [0.016, 0.832]) and, on a trend level, compared to unmanipulated (*p* = 0.066, M_Diff_ = 0.365, 95% CI [−0.018, 0.745]).

Negative expressions were more often classified as neutral when physical salience was high compared to unmanipulated (*p* = 0.013, M_Diff_ = −0.576, 95% CI [−1.056, −0.095], d = 0.36), but not compared to low (*p* = 1.00). For positive expressions, there were no significant differences (all *p*s > 0.28). A main effect for physical salience regarding misclassifications was found but was not interpreted due to the higher order interaction effect. All effect sizes for *post hoc* tests can be found in Supplementary Tables S6, S7.

#### 3.2.2 FEST-2: saturation

**Figure 3:**
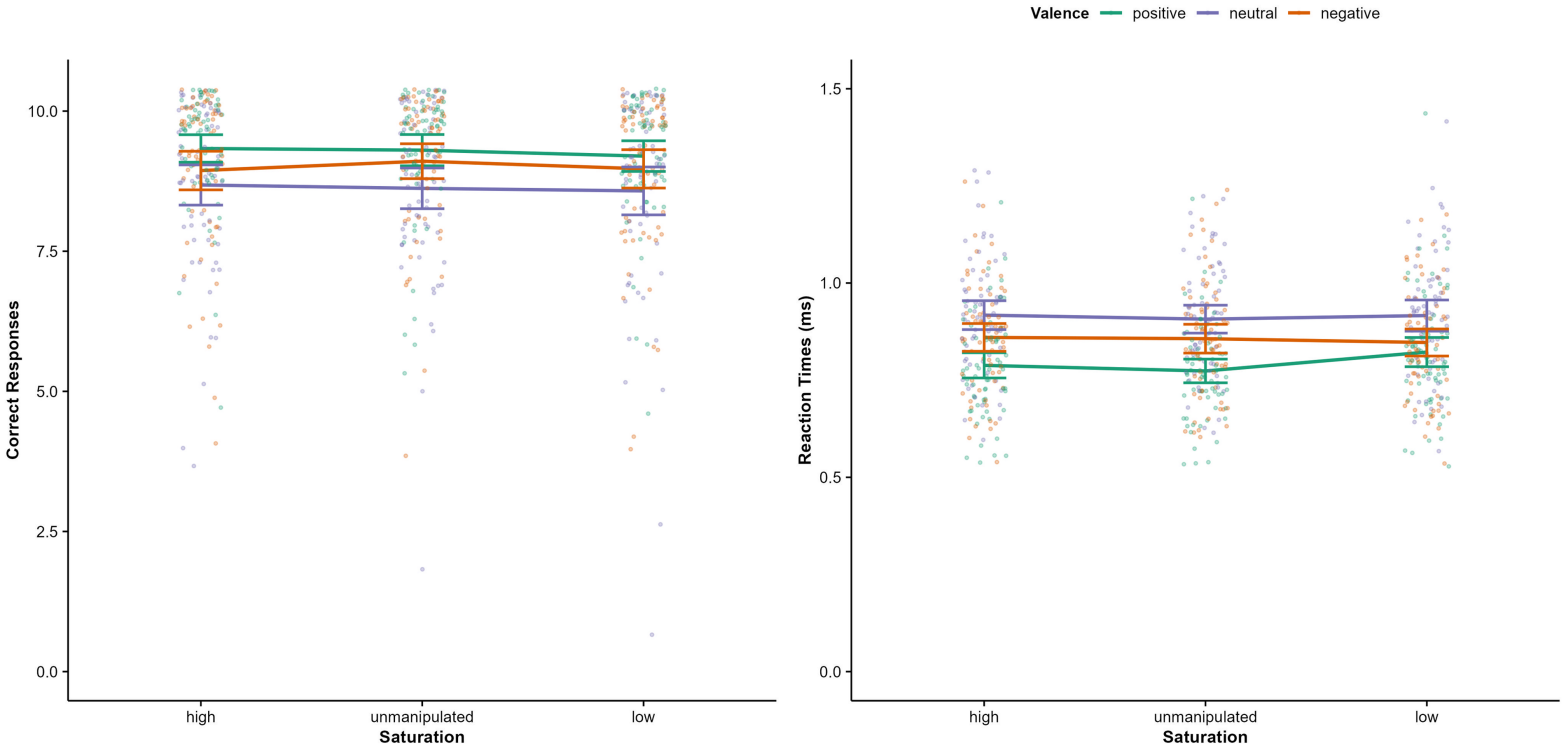
Correct responses and reaction times, separately for valence of facial expression and physical salience condition (saturation). Error bars represent 95% Confidence Intervals.

Regarding accuracy, a significant main effect for valence was found [*F*(1.756, 114.17) = 5.81, *p* = 0.006, *η*^2^*_p_* = 0.08]: Neutral faces were recognized less accurately than positive (*p* < 0.001, M_Diff_ = −0.652, 95% CI [−0.242, −1.061], d = 0.48) but not than negative faces (*p* = 0.288; see Figure 3). Accuracy for positive and negative expressions did not differ significantly (*p* = 0.406, M_Diff_ = 0.273, 95% CI[−0.170, 0.716]). No significant main effect of physical salience [*F*(2, 130) = 0.82, *p* = 0.443, *η*^2^*_p_* = 0.01], and no valence x physical salience interaction [*F*(3.01, 195.38) = 0.438, *p* = 0.726, *η*^2^*_p_* = 0.01] were found.

Regarding reaction times, there was a significant physical salience x valence interaction (see Table 2). Post-hoc comparisons revealed that for positive facial expressions, reaction times were significantly higher in the low physical salience condition compared to high (*p* = 0.011, M_Dif_ = 0.034, 95% CI [0.006, 0.062]) and unmanipulated condition (*p* < 0.001, M_Diff_ = 0.049, 95% CI [0.020, 0.077]; see Figure 3). No further post-hoc tests reached significance (all *p*s > 0.39). The significant main effect of valence and a main effect of physical salience were not interpreted due to the higher order interaction effect.

**Table 2:** Within-subjects ANOVA results for FEST-2: saturation.

Effect	*df*	*df(error)*	*F*	*η* ^2^ *_p_*	*p*
Accuracy					
Valence	1.76	114.17	5.81	0.08	0.006
Physical salience	2	130	0.82	0.01	0.443
Valence × physical salience	3.01	195.38	0.44	0.01	0.726
Reaction times					
Valence	2	130	40.99	0.39	<0.001
Physical salience	2	130	2.97	0.04	0.055
Valence × physical salience	4	260	3.55	0.05	0.008
Misclassifications					
Type	2.25	145.91	18.26	0.22	<0.001
Physical salience	2	130	0.87	0.01	0.432
Type × physical salience	5.89	382.41	1.56	0.02	0.159

No significant misclassification type x physical salience interaction [*F*(5.89, 382.41) = 1.56, *p* = 0.159, *η*^2^*_p_* = 0.02] and no main effect of physical salience on misclassifications were found, but there was a main effect of misclassification type. The most frequent misclassifications were those of negative expressions as neutral and neutral expressions as negative (all *p*s < 0.024). Other misclassifications did not differ significantly (all *p*s > = 0.066). All effect sizes for *post hoc* tests can be found in Supplementary Tables S6, S7.

### 3.3 Associations with schizotypy

Schizotypy was not significantly associated with the FEST. Neither total scores nor positive or disorganized factor scores of the O-LIFE significantly predicted negative bias (all *p*s > = 0.17, see Supplementary Table S1). Spearman correlations between difference values of each physical salience level for each valence with the O-LIFE scores are reported in Supplementary Tables S2, S3. Although some associations were found, none survived correction for multiple testing.

## 4 Discussion

We investigated whether emotion recognition impairments characteristic for schizophrenia (Kohler et al., 2003; Mier et al., 2014) can be simulated in healthy participants. To this end, novel emotion recognition tasks combining physical and emotional salience were applied. We hypothesized that high physical salience would be perceived as being congruent with emotional salience and consequently lead to faster, more accurate recognition for emotional expressions. For neutral expressions, high physical salience should lead to incongruence and therefore slower, less accurate recognition. Based on findings from SZ and schizotypy research (Kohler et al., 2003; Premkumar et al., 2008; Brown and Cohen, 2010), we further expected more misclassifications of neutral expressions for high physical salience, with a negative bias. Psychometric schizotypy was assumed to be associated with this interaction of physical and emotional salience, with positive and disorganised dimensions predicting generally more negative bias.

We found some effects of physical salience on emotion recognition. The effects differed for the contrast (FEST-1, see section 4.1) and saturation manipulations (FEST-2, see section 4.2). Contrary to our hypotheses, no significant associations with schizotypy were found.

### 4.1 Contrast affects negative and neutral expression recognition

We found an interaction of contrast and emotional salience for recognition speed. In addition, the type of misclassification differed between conditions.

For high contrast pictures, negative facial expressions were recognized faster and neutral facial expressions more slowly with small to medium effect sizes. This pattern suggests congruence effects of physical and emotional salience, i.e., when physical salience is high due to increased contrast in emotionally salient negative expressions, recognition is facilitated. However, this did not hold true for positive expressions which were not recognized faster. In agreement with the finding of higher nucleus accumbens activation for fearful than happy facial expression during final decision making (Schmidt et al., 2019), this inconsistency may be explained by a greater emotional salience of negative, threatening faces (Palermo and Rhodes, 2007) and, therefore, a stronger congruence effect of physical and emotional salience. Another potential explanation may lie in the chosen manipulation of physical salience. By analysing the frequency spectra of emotional facial expressions, Hedger et al. (2015) showed that fearful expressions have higher effective contrast than neutral faces, which may result in a processing advantage of fear or threat. Increased contrast may have thus been perceived as congruent for negative, but not positive expressions. However, others argue that the higher contrast of fearful faces is often a result of image normalisation (Webb and Hibbard, 2019; Webb et al., 2020). Thus, enhancing the contrast in our study might have increased salience disproportionally for fearful faces.

For neutral, emotionally less salient faces, increased contrast may have led to incongruence of physical and emotional salience and thus slower processing. This suggests that with high physical salience, more emotional significance might be attributed to neutral expressions. We propose that this might represent a simulation of aberrant salience processing: Resembling aberrant salience in psychosis (Kapur, 2003; Kapur et al., 2005), content-inappropriate perceived salience might have led to aberrant emotional salience attribution to neutral stimuli. In other words: When contrast is high for neutral faces, people may conflate physical and emotional salience, leading to interference and slower processing. In a model of social-cognitive deficits in SZ (Mier and Kirsch, 2017), chaotic dopaminergic signalling is considered to evoke hypersalience in response to neutral or irrelevant social stimuli, i.e., social stimuli without emotional salience. In healthy participants, an increase in physical salience may have simulated this hypersalience. Neutral faces were not only recognized more slowly, but also more often misclassified as negative. Although these effects were rather small, aberrant salience seems to lead to a negative bias rather than a general emotional bias, since increased contrast did not affect misclassifications as positive. This pattern of misclassification has also been found in SZ (Mier et al., 2014) and schizotypy (Brown and Cohen, 2010), suggesting that perceived hypersalience is associated with misattribution of negative valence rather than general emotionality. Again, contrast as a salience manipulation may have influenced these findings, as high contrast may be more congruent with negative valence (Hedger et al., 2015). Negative faces were also more often misclassified as neutral when physical salience was high, suggesting that high contrast generally impairs the distinction of negative and neutral faces. However, this was only found compared to the unmanipulated but not the low physical salience condition. Thus, the effect may rather reflect higher task difficulty due to the manipulation itself. Previous studies argue that aberrant salience might lead to an emotional rather than negative bias and that the attribution of negative valence may be explained by negative affect or anxiety, commonly present in SZ (Schmidt et al., 2019). To investigate for this possibility, exploratory analyses revealed an association of the negative bias with the obsession compulsion, the phobic anxiety and, on a trend level, the anxiety scale of the BSI. While these associations were not specific for the high salience condition (see Supplementary Tables S4, S5), and did not survive correction for multiple testing, anxiety might have an influence on the occurrence of the negative bias. Thus, future studies should further focus on the impact of anxiety on biased perception of social stimuli. This might be particularly interesting in clinical samples of patients with SZ who suffer from anxieties related to the content of their delusions, but also in patients with different anxiety disorders. This is especially relevant with regard to social anxiety. While no significant associations were found with the interpersonal sensitivity scale of the BSI in our study, other researchers found enhanced sensitivity to negative facial expressions (Rossignol et al., 2013), and a negative bias in social anxiety (Clark and McManus, 2002; Günther et al., 2021). Since social anxiety is assumed to contribute to disorganisation in schizotypy (Mason et al., 1995; Premkumar and Kumari, 2022) it might have an impact on biased perceptions in schizophrenia spectrum disorders. Further, since social anxiety is characterized by enhanced attention to possible social threats (Mogg and Bradley, 2002), social anxiety might provide a cognitive explanation for a negative bias in emotion recognition even across diagnostic groups.

### 4.2 Saturation affects positive expression recognition

Positive emotions were recognized more slowly when saturation was decreased compared to unmanipulated and increased, suggesting an incongruence effect of physical and positive emotional salience. However, these findings may represent colour valence rather than salience effects. Bright and more saturated colours have been associated with positive valence (Wilms and Oberfeld, 2018). In faces, especially increased facial redness is associated with health and attractiveness (Thorstenson, 2018), suggesting positive evaluation, while desaturated faces may represent a physiological fear reaction (“turning pale”; Montoya et al., 2005). Alternatively, however, high accuracy rates in the saturation condition may have reduced physical salience effects.

### 4.3 Associations with schizotypy

Based on previous findings regarding aberrant salience, and impaired emotion recognition, as well as the association of both in SZ (Kohler et al., 2003; Heinz and Schlagenhauf, 2010; Comparelli et al., 2020) and schizotypy (Cowan et al., 2019; Raballo et al., 2019), we expected an association of schizotypy with an increased effect of physical salience on emotion recognition, and more negative bias. However, no significant associations with schizotypy were found.

While several studies found emotion recognition deficits in schizotypy (for a review, see Giakoumaki, 2016), findings are inconsistent regarding schizotypy dimensions, reporting associations with negative (Dawes et al., 2021), disorganized (Brown and Cohen, 2010), or positive (Germine and Hooker, 2011) schizotypy. Altered recognition of neutral (Brown and Cohen, 2010), positive (Williams et al., 2007) or negative expressions (Dawes et al., 2021) has been found. Similar to our study, Jahshan and Sergi (2007) found no social cognition impairment in schizotypy and concluded that psychometric schizotypes are not impaired in social cognition. Alfimova et al. (2009) found that while facial emotion recognition deficits were present in genetic risk, they did not correlate with schizotypy scores. Sample characteristics such as questionnaires used, high risk vs. general population or student samples, as well as task characteristics may add to these inconsistent findings (Cowan et al., 2019).

Despite a lack of association with psychometric schizotypy, our findings point to aberrant salience processing as a promising framework to explain altered emotion recognition. In healthy participants, increased physical salience by enhanced contrast impacted the recognition of negative and neutral facial expressions and led to more negative bias. People who show a negative bias in this task do not show more schizotypal traits, but a similar pattern of impaired emotion recognition has been found in SZ (Mier et al., 2014). Additionally, hypersalience in response to neutral stimuli has been demonstrated in SZ (Holt et al., 2006) and has been proposed to explain delusion formation (Kapur, 2003). On a neural level, altered brain activation during neutral or ambiguous face recognition has been found in regions implicated in salience and intention processing (Seiferth et al., 2008; Schmidt et al., 2019; Yan et al., 2020). Moreover, reduced signalling in the dopaminergic substantia nigra and ventral tegmental area was found for different types of salience (negative emotional, task-related, novelty) in psychosis patients, suggesting a general salience processing deficit (Knolle et al., 2018). Psychosis patients also showed smaller activation differences in response to salient compared to non-salient stimuli, potentially reflecting hypersalience of non-salient (emotional and non-emotional) stimuli (Knolle et al., 2018).

## 5 Limitations

A limitation of the present study is the restricted comparability of the two tasks. The contrast stimuli as opposed to the saturation stimuli were in greyscale and with reduced emotional intensity of the facial expressions, increasing task difficulty which is reflected in lower accuracy rates for the contrast stimuli. Differences within each stimulus set provide partial support for differential effects of physical salience modulation, and the impact of different salience manipulations should be carefully assessed in future studies. Specifically, the impact of changes in spatial frequency should be further investigated. Contrast, but not saturation may have altered the spatial frequency of stimuli and thus influenced emotion perception. Previous findings on spatial frequency in emotion recognition have been inconsistent (Cassidy et al., 2021; Jennings et al., 2017; Kumar and Srinivasan, 2011). Future studies should apply facial stimuli that are restricted to the face to avoid possible influences of changes in contrast/saturation in the hair area, as well as the skin line. In addition, using a face-in-the-crowd paradigm, during which salient and non-salient faces are presented simultaneously, smaller effects may be detected than in our task in which salience is compared in the course of the experiment. Further, eye-tracking could be applied to study the influence of the contrast and saturation manipulations on facial feature processing and bottom-up orienting.

Additionally, task difficulty might vary between emotional expressions: Positive faces were generally recognized faster and more accurately than negative and neutral faces, replicating previous findings (Calvo and Nummenmaa, 2008), while neutral and negative expressions are more difficult to differentiate (Leppanen and Hietanen, 2004). This could lead to ceiling effects for positive expressions and a stronger effect of physical salience on negative bias. Varying task difficulty across physical salience manipulations could further clarify the interaction of physical and emotional salience. Adding other emotional expressions such as angry, sad or surprised faces could explore if the interaction is specific to valence rather than to the fearful expressions used in our study. Our stimulus material and our participants were mainly Caucasian. An ethnically diverse stimulus set, and sample could improve generality, especially as culture has been shown to affect basic face processing on a neural level (Yan et al., 2019). Further, our student sample scored relatively low on schizotypy dimensions compared to studies with larger German samples (Grant et al., 2013). Hence, we had restricted variance in our study and may have failed to detect correlations with more pronounced schizotypal traits.

Future studies should employ extreme-groups designs or include clinical schizotypy, participants with an at-risk mental state, or schizophrenia populations. Additionally, aberrant salience during emotion recognition could represent an endophenotype (Yan et al., 2020). Thus, physiological measures should be included, because deficits may be correlated with subclinical schizotypy on a neural rather than behavioural level, also due to adaptive compensation (Seiferth et al., 2008; Knolle et al., 2018). Further, emotion recognition impairments, as well as a negative bias were found in several mental disorders (e.g., borderline personality disorder (Fenske et al., 2015), posttraumatic stress disorder (Couette et al., 2020), obsessive-compulsive disorder (Aigner et al., 2007), social anxiety (Kivity and Huppert, 2016), and panic disorder (Bottinelli et al., 2021)). However, to our knowledge, the negative bias in these disorders has not been related to aberrant salience. Thus, future studies might apply the FEST to compare patients with different mental disorders to explore a potentially differential influence of salience on emotion recognition.

## 6 Conclusion

Overall, our findings suggest that changes in physical salience can influence facial emotion recognition depending on the matching between emotional and physical salience. The results show differential effects of physical salience (contrast and saturation) on recognition speed for positive, negative and neutral facial expressions. Negative expression processing was facilitated by high physical salience (contrast). Positive expression processing was impaired by low physical salience (saturation). Neutral expressions were recognized more slowly when contrast was high and were also more often misclassified as negative, suggesting that high physical salience induced an increased attribution of emotional salience. Thus, we propose that the FEST may be suitable to simulate aberrant salience processing during emotion recognition in healthy participants. Although these results were not associated with schizotypy scores, aberrant salience might be a promising framework to explain alterations and biases in emotion recognition in psychosis. Further research should include clinical populations to gain insight on how experimentally induced aberrant salience may explain social-cognitive deficits and biases in mental disorders.

## Data Availability

The datasets presented in this study can be found in online repositories. The names of the repository/repositories and accession number(s) can be found at: https://osf.io/thspd/?view_only=40a7366547314790aca5879c7e84bf4c.

## Footnotes

^1^To our knowledge, emotional and motivational salience have not been strictly theoretically differentiated but are used depending on the field of study. Since our study focuses on emotional facial expressions, we will use the term emotional salience from here on.
