# Supplementary material for: How to elicit a negative bias? Manipulating contrast and saturation with the facial emotion salience task
Source: Front Psychol. 2024 Aug 29;15:1284595. doi: 10.3389/fpsyg.2024.1284595 (PMC11390599; doi:10.3389/fpsyg.2024.1284595)
Supplement: Supplementary file 1 [file Data_Sheet_1.docx]

**Supplementary Materials**

**Experimental tasks and questionnaires**

These experimental tasks will be presented elsewhere:

The Emotional Visual Scene Recognition Task (adapted from Schettino et al., 2012) is a progressive unfolding tasks in which participants are asked to memorize and match pleasant, unpleasant, or neutral scenes from the International Affective Picture System (Lang and Bradley, 2007). It consists of 120 trials with the following procedure: After a fixation cross, a greyscale scene is shown for 1500ms and then masked for 2000ms. After another fixation cross, a blurry version of the same scene, of a new scene or of a scrambled scene is presented for 500ms, followed by a blank screen for 250ms. This is repeated up to 5 times to progressively unveil the image, each step adding high and low spatial frequency information. Participants are asked to press a button as soon as they recognize if the scene is the same or different and then indicate their decision (same/different). After another fixation cross, they are asked to rate the pictures in valence and arousal using 9-point self-assessment manikins.

The Trust Game (adapted from Erkic et al., 2018), is an interaction game that measures trust in interpersonal interactions. First, participants view four images of a person with a neutral facial expression (trustee) and rate their sympathy. Second, they are asked to invest between 10 and 90 points in the trustees, which is multiplied by 3 and can be partially returned to the participant. After each round, given and received points are displayed for 10000ms. Third, participants rate sympathy and fairness of the trustees again. Each rating is given on a 9-point-scale. Points are returned in cents to participants as compensation,

The Beads task (Huq et al., 1988) in a one-round version is used to measure the jumping to conclusions bias. One of two jars containing 80 red and 20 blue beads, or 20 red and 80 blue beads each is selected without the participant’s knowledge. Participants are asked to identify the chosen jar by drawing beads from the jar which are then presented. After each bead, they are asked to either decide on the jar if they think they identified the correct jar or draw another bead. Previously drawn beads are presented in the upper right corner of the screen.

Further questionnaires that will be presented elsewhere include the Emotion Regulation Questionnaire (Abler and Kessler, 2009), Autism-Spectrum Questionnaire (Freitag et al., 2007), Impulsive Behaviour Scale (Kovaleva et al., 2012), Behavioural Inhibition System/Behavioral Activation System Scales (Strobel et al., 2001), Interpersonal Reactivity Index (Paulus, 2009), Schizotypal Personality Questionnaire (Klein et al., 1997), Social Phobia and Anxiety Questionnaire (Fydrich, 2002) and Short Scale for Interpersonal Trust (Beierlein, 2014).

| **TABLE S1 Coefficients of multiple regressions of schizotypy scores on negative bias** | | | | | | | | |
| --- | --- | --- | --- | --- | --- | --- | --- | --- |
| **misclassification**  **type** | | **unstandardised**  **coefficients** | | **standardised**  **coefficients** | ***t*** | ***p*** | **95.0% CI** | |
|  |  | **B** | **Std. Error** | **Beta** |  |  | **lower bound** | **upper bound** |
| Contrast | | | | | | | | |
| positive as negative | (Constant) | 2.961 | 0.969 |  | 3.057 | 0.003 | 1.025 | 4.897 |
|  | OLIFETotal | -0.054 | 0.096 | -0.253 | -0.565 | 0.574 | -0.247 | 0.138 |
|  | UnEx | 0.126 | 0.162 | 0.247 | 0.778 | 0.440 | -0.198 | 0.450 |
|  | CogDis | 0.019 | 0.133 | 0.037 | 0.143 | 0.887 | -0.247 | 0.285 |
| neutral as negative | (Constant) | 8.134 | 1.643 |  | 4.949 | < 0.001 | 4.848 | 11.419 |
|  | OLIFETotal | -0.202 | 0.164 | -0.548 | -1.237 | 0.221 | -0.529 | 0.125 |
|  | UnEx | 0.355 | 0.275 | 0.405 | 1.288 | 0.202 | -0.196 | 0.905 |
|  | CogDis | 0.285 | 0.226 | 0.325 | 1.263 | 0.211 | -0.196 | 0.736 |
| saturation | | | | | | | | |
| positive as negative | (Constant) | -0.015 | 0.512 |  | -0.29 | 0.977 | -1.038 | 1.091 |
|  | OLIFETotal | 0.057 | 0.051 | 0.478 | 1.110 | 0.271 | -0.045 | 0.158 |
|  | UnEx | -0.002 | 0.086 | -0.008 | -0.027 | 0.978 | -0.174 | 0.169 |
|  | CogDis | -0.089 | 0.070 | -0.318 | -1.270 | 0.209 | -0.230 | 0.051 |
| neutral as negative | (Constant) | 1.317 | 1.393 |  | 0.945 | 0.348 | -1.468 | 4.101 |
|  | OLIFETotal | 0.173 | 0.139 | 0.553 | 1.251 | 0.216 | -0.104 | 0.450 |
|  | UnEx | -0.168 | 0.233 | -0.225 | -0.718 | 0.475 | -0.634 | 0.299 |
|  | CogDis | -0.207 | 0.191 | -0.278 | -1.081 | 0.284 | -0.589 | 0.175 |

*Note.* Dependent Variable: number of misclassifications, UnEx = Unusual Experiences. CogDis = Cognitive Disorganisation.

| **TABLE S2 Correlations of difference values with schizotypy scores for FEST- 1 (contrast)** | | | | | | | | | | |
| --- | --- | --- | --- | --- | --- | --- | --- | --- | --- | --- |
|  | | **O-LIFE Total** | **UnEx** | | | **CogDis** | **IntAn** | | **ImpNon** | |
| correct responses | | | | | | | | | | |
| pos_p - pos_m | ρ | -0.035 | | 0.087 | -0.094 | | | -0.206 | | 0.032 |
|  | Sig. | 0.780 | | 0.486 | 0.452 | | | 0.097 | | 0.801 |
| pos_p - pos_s | ρ | -0.163 | | -0.040 | -0.170 | | | -0.203 | | -0.014 |
|  | Sig. | 0.191 | | 0.752 | 0.174 | | | 0.102 | | 0.908 |
| pos_s - pos_m | ρ | 0.093 | | 0.118 | 0.045 | | | -0.027 | | 0.036 |
|  | Sig. | 0.460 | | 0.347 | 0.721 | | | 0.831 | | 0.777 |
| ne_p - ne_m | ρ | 0.009 | | 0.136 | -0.051 | | | -0.064 | | 0.027 |
|  | Sig. | 0.944 | | 0.278 | 0.685 | | | 0.612 | | 0.829 |
| ne_p - ne_s | ρ | 0.036 | | 0.096 | -0.057 | | | -0.074 | | 0.171 |
|  | Sig. | 0.776 | | 0.443 | 0.652 | | | 0.555 | | 0.171 |
| ne_s - ne_m | ρ | 0.000 | | 0.024 | 0.032 | | | 0.036 | | -0.100 |
|  | Sig. | 0.999 | | 0.848 | 0.797 | | | 0.771 | | 0.426 |
| neg_p - neg_m | ρ | -0.151 | | -0.200 | -0.160 | | | 0.047 | | -0.053 |
|  | Sig. | 0.226 | | 0.108 | 0.199 | | | 0.707 | | 0.672 |
| neg_p - neg_s | ρ | -0.166 | | -0.203 | 0.004 | | | -0.240 | | -0.145 |
|  | Sig. | 0.184 | | 0.103 | 0.976 | | | 0.052 | | 0.246 |
| neg_s - neg_m | ρ | 0.007 | | -0.019 | -0.144 | | | **.287^*^** | | 0.075 |
|  | Sig. | 0.955 | | 0.881 | 0.247 | | | **0.019** | | 0.552 |

|  | | **O-LIFE Total** | | **UnEx** | | | **CogDis** | | **IntAn** | | **ImpNon** |
| --- | --- | --- | --- | --- | --- | --- | --- | --- | --- | --- | --- |
| reaction times | | | | | | | | | | | |
| pos_p - pos_m rt | ρ | | -0.052 | | 0.003 | -0.004 | | -0.080 | | -0.062 | |
|  | Sig. | | 0.678 | | 0.984 | 0.976 | | 0.522 | | 0.620 | |
| pos_p - pos_s rt | ρ | | -0.198 | | -0.161 | -0.097 | | -0.204 | | -0.069 | |
|  | Sig. | | 0.111 | | 0.196 | 0.438 | | 0.100 | | 0.582 | |
| pos_s - pos_m rt | ρ | | 0.149 | | 0.156 | 0.128 | | 0.088 | | 0.075 | |
|  | Sig. | | 0.232 | | 0.212 | 0.305 | | 0.482 | | 0.550 | |
| ne_p - ne_m rt^a^ | ρ | | 0.030 | | 0.042 | 0.052 | | -0.077 | | -0.045 | |
|  | Sig. | | 0.815 | | 0.737 | 0.680 | | 0.540 | | 0.719 | |
| ne_p - ne_s rt^a^ | ρ | | 0.013 | | 0.082 | -0.074 | | -0.160 | | 0.155 | |
|  | Sig. | | 0.917 | | 0.518 | 0.559 | | 0.203 | | 0.217 | |
| ne_s - ne_m rt | ρ | | 0.060 | | -0.045 | 0.161 | | 0.046 | | -0.103 | |
|  | Sig. | | 0.634 | | 0.719 | 0.197 | | 0.712 | | 0.408 | |
| neg_p - neg_m rt | ρ | | 0.119 | | 0.051 | 0.122 | | 0.106 | | -0.003 | |
|  | Sig. | | 0.339 | | 0.687 | 0.330 | | 0.397 | | 0.980 | |
| neg_p - neg_s rt^b^ | ρ | | 0.044 | | 0.204 | -0.013 | | -0.105 | | 0.059 | |
|  | Sig. | | 0.728 | | 0.106 | 0.917 | | 0.411 | | 0.645 | |
| neg_s - neg_m rt^b^ | ρ | | 0.057 | | -0.124 | 0.131 | | 0.104 | | -0.031 | |
|  | Sig. | | 0.657 | | 0.331 | 0.302 | | 0.414 | | 0.809 | |
| *Note.* N = 66. neg = negative. pos = positive. ne = neutral. p = high salience condition. s = unmanipulated salience condition. m = low salience condition rt = reaction time. | | | | | | | | | | | |
| ^a^ N = 65. ^b^ N = 64  * Correlation is significant at the 0.05 level | | | | | | | | | | | |

| **TABLE S3 Correlations of difference values with schizotypy scores for FEST-2 (saturation)** | | | | | | |  |
| --- | --- | --- | --- | --- | --- | --- | --- |
|  | | **O-LIFE Total** | **UnEx** | **CogDis** | **IntAn** | **ImpNon** |  |
| correct responses | | | | | | |  |
| pos_p - pos_m | ρ | -0.132 | -0.036 | -0.114 | 0.011 | -0.200 | |
|  | Sig. | 0.290 | 0.776 | 0.362 | 0.928 | 0.108 | |
| pos_p - pos_s | ρ | 0.003 | 0.093 | 0.042 | -0.168 | 0.052 | |
|  | Sig. | 0.982 | 0.456 | 0.737 | 0.177 | 0.678 | |
| pos_s - pos_m | ρ | -0.170 | -0.092 | -0.203 | 0.108 | **-.272^*^** | |
|  | Sig. | 0.173 | 0.464 | 0.102 | 0.388 | **0.027** | |
| ne_p - ne_m | ρ | 0.081 | 0.111 | -0.060 | 0.102 | 0.099 | |
|  | Sig. | 0.517 | 0.375 | 0.630 | 0.413 | 0.428 | |
| ne_p - ne_s | ρ | -0.057 | -0.062 | -0.034 | -0.093 | 0.010 | |
|  | Sig. | 0.649 | 0.621 | 0.785 | 0.458 | 0.939 | |
| ne_s - ne_m | ρ | 0.105 | 0.118 | -0.044 | 0.153 | 0.098 | |
|  | Sig. | 0.402 | 0.347 | 0.728 | 0.219 | 0.435 | |
| neg_p - neg_m | ρ | -0.171 | -0.144 | -0.086 | -0.054 | **-.268^*^** | |
|  | Sig. | 0.170 | 0.247 | 0.493 | 0.668 | **0.030** | |
| neg_p - neg_s | ρ | -0.224 | **-.243^*^** | -0.159 | -0.219 | -0.006 | |
|  | Sig. | 0.071 | **0.050** | 0.202 | 0.077 | 0.965 | |
| neg_s - neg_m | ρ | -0.001 | 0.047 | 0.009 | 0.090 | -0.224 | |
|  | Sig. | 0.991 | 0.710 | 0.945 | 0.474 | 0.071 | |

|  | | **O-LIFE Total** | **UnEx** | | **CogDis** | | **IntAn** | | **ImpNon** | |  |
| --- | --- | --- | --- | --- | --- | --- | --- | --- | --- | --- | --- |
| reaction times | | | | | | | | | | |  |
| pos_p - pos_m rt | ρ | -0.107 | | -0.102 | | -0.167 | | 0.012 | | 0.026 | |
|  | Sig. | 0.392 | | 0.417 | | 0.179 | | 0.923 | | 0.835 | |
| pos_p - pos_s rt | ρ | **.295^*^** | | **.250^*^** | | 0.088 | | 0.214 | | **.391^**^** | |
|  | Sig. | **0.016** | | **0.043** | | 0.484 | | 0.085 | | **0.001** | |
| pos_s - pos_m rt | ρ | **-.365^**^** | | **-.319^**^** | | **-.272^*^** | | -0.164 | | **-.307^*^** | |
|  | Sig. | **0.003** | | **0.009** | | **0.027** | | 0.188 | | **0.012** | |
| ne_p - ne_m rt | ρ | -0.100 | | -0.060 | | -0.022 | | -0.154 | | -0.074 | |
|  | Sig. | 0.423 | | 0.634 | | 0.863 | | 0.217 | | 0.557 | |
| ne_p - ne_s rt | ρ | -0.077 | | -0.016 | | -0.017 | | -0.010 | | -0.131 | |
|  | Sig. | 0.537 | | 0.897 | | 0.891 | | 0.936 | | 0.296 | |
| ne_s - ne_m rt | ρ | -0.122 | | -0.127 | | -0.073 | | -0.204 | | -0.015 | |
|  | Sig. | 0.331 | | 0.309 | | 0.558 | | 0.100 | | 0.902 | |
| neg_p - neg_m rt | ρ | -0.131 | | -0.151 | | -0.016 | | -0.067 | | -0.117 | |
|  | Sig. | 0.294 | | 0.226 | | 0.896 | | 0.594 | | 0.350 | |
| neg_p - neg_s rt | ρ | **-.253^*^** | | -0.134 | | -0.166 | | -0.087 | | **-.258^*^** | |
|  | Sig. | **0.040** | | 0.284 | | 0.182 | | 0.490 | | **0.037** | |
| neg_s - neg_m rt | ρ | 0.157 | | 0.001 | | 0.157 | | 0.122 | | 0.142 | |
|  | Sig. | 0.208 | | 0.995 | | 0.209 | | 0.330 | | 0.256 | |
| *Note.* N = 66*.* neg = negative, pos = positive, ne = neutral, p = high salience condition, s = unmanipulated salience condition, m = low salience condition rt = reaction time. | | | | | | | | | | |  |
| * Correlation is significant at the 0.05 level | | | | | | | | | | |  |

**Associations with the Brief Symptom Inventory (BSI)**

The BSI (Franke, 2000) is a short 53-item version of the Symptom Checklist-90-revised (Derogatis, 1975) that measures nine dimensions of psychological symptoms on a four-point intensity rating scale: Somatization, Obsession-Compulsion, Interpersonal Sensitivity, Depression, Anxiety, Hostility, Phobic anxiety, Paranoid Ideation and Psychoticism. Internal consistency in our sample was good for the Depression (α = .87) and Obsession Compulsion scales (α = .81), acceptable for the Interpersonal Sensitivity (α = .77), Anxiety (α = .73), Somatization (α = .73), and Psychoticism scales (α = .71), but questionable for the Hostility (α = .69) and Paranoid Ideation scales (α = .64). Welch tests were used to explore if our sample differed from a norm student sample (N = 589; Franke, 2000). Exploratively, we calculated Spearman correlations of the BSI subscales with the number of misclassifications of neutral and positive facial expressions as negative (i.e., negative bias).

Mean scores for the subscales were .28 (SD = .36) for Somatization, .82 (SD = .68) for Obsession Compulsion, .50 (SD = .57) for Interpersonal Sensitivity, .43 (SD = .60) for Depression, .48 (SD = .54) for Anxiety, .37 (SD = .49) for Hostility, .36 (SD = .55) for Phobic Anxiety, .34 (SD = .45) for Paranoid Ideation, and .30 (SD = .47) for Psychoticism. Compared to the student norm sample, Interpersonal Sensitivity, Hostility and Paranoid Ideation scores were significantly lower, all other subscales did not differ significantly. Associations of the negative bias with the obsession compulsion, phobic anxiety and, on a trend level, with the anxiety scale of the BSI were found for the contrast condition (see table S4), and with interpersonal sensitivity for the low saturation condition (table S5).

| **TABLE S4 Correlation of BSI Depression and Anxiety subscales with misclassifications (FEST-1: contrast)** | | | | | | | | | | | |
| --- | --- | --- | --- | --- | --- | --- | --- | --- | --- | --- | --- |
| **misclassification type** | |  | **Somatization** | **Obsession Compulsion** | **Interpersonal Sensitivity** | **Depression** | **Anxiety** | **Hostility** | **Phobic Anxiety** | **Paranoid Ideation** | **Psychoticism** |
| positive as negative (all) | | ρ | 0.045 | 0.053 | 0.000 | 0.023 | -0.098 | 0.032 | -0.068 | 0.117 | 0.208 |
|  |  | Sig. | 0.721 | 0.670 | 0.998 | 0.854 | 0.435 | 0.802 | 0.587 | 0.350 | 0.094 |
| positive as negative (p) | | ρ | -0.023 | 0.098 | -0.041 | 0.046 | -0.026 | -0.015 | 0.057 | 0.055 | 0.234 |
|  |  | Sig. | 0.856 | 0.434 | 0.744 | 0.713 | 0.837 | 0.905 | 0.652 | 0.662 | 0.058 |
| positive as negative (s) | | ρ | 0.074 | -0.039 | -0.041 | -0.127 | -0.175 | 0.001 | -0.151 | 0.032 | 0.055 |
|  |  | Sig. | 0.553 | 0.754 | 0.743 | 0.308 | 0.159 | 0.992 | 0.225 | 0.796 | 0.663 |
| positive as negative (m) | | ρ | 0.096 | 0.063 | 0.079 | 0.079 | 0.030 | 0.155 | -0.090 | 0.204 | 0.184 |
|  |  | Sig. | 0.445 | 0.616 | 0.528 | 0.529 | 0.813 | 0.214 | 0.472 | 0.101 | 0.140 |
| neutral as negative (all) | | ρ | 0.014 | **.291^*^** | -0.014 | 0.106 | 0.205 | 0.184 | **.291^*^** | 0.023 | 0.185 |
|  |  | Sig. | 0.909 | **0.018** | 0.913 | 0.397 | 0.099 | 0.138 | **0.018** | 0.854 | 0.138 |
| neutral as negative (p) | | ρ | 0.058 | 0.196 | -0.008 | 0.105 | 0.238 | 0.153 | 0.232 | -0.032 | 0.168 |
|  |  | Sig. | 0.645 | 0.114 | 0.947 | 0.403 | 0.054 | 0.219 | 0.061 | 0.800 | 0.177 |
| neutral as negative (s) | | ρ | -0.011 | 0.232 | -0.052 | 0.081 | 0.170 | 0.131 | **.280^*^** | 0.088 | 0.155 |
|  |  | Sig. | 0.930 | 0.061 | 0.676 | 0.517 | 0.173 | 0.294 | **0.023** | 0.480 | 0.214 |
| neutral as negative (m) | | ρ | 0.027 | **.246^*^** | -0.047 | 0.047 | 0.051 | 0.158 | 0.192 | 0.028 | 0.121 |
|  |  | Sig. | 0.830 | **0.047** | 0.706 | 0.706 | 0.684 | 0.206 | 0.123 | 0.824 | 0.332 |
| *Note.* N = 66. BSI = Brief Symptom Inventory. p = low salience condition, s = unmanipulated salience condition, m = low salience condition. | | | | | | | | | | | |

| **TABLE S5 Correlation of BSI Depression and Anxiety subscales with misclassifications (FEST-2: saturation)** | | | | | | | | | | | |
| --- | --- | --- | --- | --- | --- | --- | --- | --- | --- | --- | --- |
| **misclassification type** | |  | **Somatization** | **Obsession Compulsion** | **Interpersonal Sensitivity** | **Depression** | **Anxiety** | **Hostility** | **Phobic Anxiety** | **Paranoid Ideation** | **Psychoticism** |
| positive as negative (all) | | ρ | 0.230 | 0.004 | 0.056 | -0.025 | -0.044 | 0.000 | 0.040 | -0.035 | 0.178 |
|  |  | Sig. | 0.063 | 0.975 | 0.657 | 0.840 | 0.729 | 0.998 | 0.752 | 0.777 | 0.152 |
| positive as negative (p) | | ρ | 0.204 | 0.136 | -0.045 | 0.116 | -0.020 | 0.059 | 0.048 | 0.020 | 0.187 |
|  |  | Sig. | 0.101 | 0.278 | 0.717 | 0.354 | 0.873 | 0.636 | 0.704 | 0.876 | 0.132 |
| positive as negative (s) | | ρ | -0.006 | 0.137 | -0.015 | -0.082 | 0.018 | -0.010 | -0.055 | -0.032 | 0.105 |
|  |  | Sig. | 0.965 | 0.272 | 0.908 | 0.515 | 0.883 | 0.936 | 0.660 | 0.800 | 0.403 |
| positive as negative (m) | | ρ | 0.113 | -0.175 | 0.054 | -0.096 | -0.104 | -0.177 | 0.048 | -0.053 | 0.117 |
|  |  | Sig. | 0.368 | 0.159 | 0.668 | 0.441 | 0.407 | 0.154 | 0.699 | 0.671 | 0.351 |
| neutral as negative (all) | | ρ | -0.049 | 0.111 | -0.228 | -0.039 | -0.142 | -0.049 | -0.003 | -0.134 | 0.058 |
|  |  | Sig. | 0.696 | 0.377 | 0.065 | 0.758 | 0.257 | 0.694 | 0.980 | 0.285 | 0.646 |
| neutral as negative (p) | | ρ | -0.124 | 0.054 | -0.217 | -0.019 | -0.175 | -0.117 | 0.029 | -0.125 | 0.117 |
|  |  | Sig. | 0.321 | 0.667 | 0.080 | 0.882 | 0.160 | 0.351 | 0.817 | 0.318 | 0.347 |
| neutral as negative (s) | | ρ | -0.026 | 0.156 | -0.142 | -0.030 | 0.004 | 0.040 | 0.071 | 0.013 | 0.031 |
|  |  | Sig. | 0.834 | 0.212 | 0.257 | 0.810 | 0.974 | 0.752 | 0.571 | 0.919 | 0.803 |
| neutral as negative (m) | | ρ | 0.125 | 0.100 | **-.257^*^** | -0.030 | -0.149 | 0.045 | -0.046 | -0.181 | -0.033 |
|  |  | Sig. | 0.318 | 0.424 | **0.037** | 0.813 | 0.231 | 0.720 | 0.712 | 0.146 | 0.790 |
| *Note.* N = 66. BSI = Brief Symptom Inventory. p = high salience condition, s = unmanipulated salience condition, m = low salience condition. | | | | | | | | | | | |

| **TABLE S6 Effect sizes of post hoc tests of differences between valence and physical salience conditions** | | | | |
| --- | --- | --- | --- | --- |
|  | **N** | ***d*** | **N** | ***d*** |
| Correct responses | contrast | | saturation | |
| positive - neutral | 66 | 0.05 | 66 | 0.48 |
| positive - negative | 66 | 0.40 | 66 | 0.19 |
| neutral - negative | 66 | 0.29 | 66 | 0.21 |
| high - unmanipulated | 66 | 0.13 | 66 | 0.04 |
| high - low | 66 | 0.23 | 66 | 0.12 |
| unmanipulated - low | 66 | 0.36 | 66 | 0.15 |
| Reaction times | contrast | | saturation | |
| positive - neutral | 63 | 1.09 | 66 | 1.05 |
| positive - negative | 63 | 0.82 | 66 | 0.66 |
| neutral - negative | 63 | 0.25 | 66 | 0.52 |
| high - unmanipulated | 63 | 0.01 | 66 | 0.16 |
| high - low | 63 | 0.11 | 66 | 0.13 |
| unmanipulated - low | 63 | 0.09 | 66 | 0.32 |
| positive: high - unmanipulated | 63 | 0.09 | 66 | 0.19 |
| positive: high - low | 63 | 0.25 | 66 | 0.37 |
| positive: unmanipulated - low | 63 | 0.18 | 66 | 0.52 |
| neutral: high - unmanipulated | 63 | 0.44 | 66 | 0.11 |
| neutral: high - low | 63 | 0.52 | 66 | 0.01 |
| neutral: unmanipulated - low | 63 | 0.05 | 66 | 0.10 |
| negative: high - unmanipulated | 63 | 0.35 | 66 | 0.03 |
| negative: increased - low | 63 | 0.39 | 66 | 0.14 |
| negative: unmanipulated - low | 63 | 0.02 | 66 | 0.10 |

*Note*. d = effect size (Cohen’s d).

| **TABLE S7 Effect sizes of post hoc tests of differences between misclassifications** | | | | |
| --- | --- | --- | --- | --- |
| **Misclassifications** | **N** | ***d*** | **N** | ***d*** |
|  | contrast | | saturation | |
| Positive as negative |  |  |  |  |
| high - unmanipulated | 66 | 0.03 | 66 | 0.14 |
| high - low | 66 | 0.14 | 66 | 0.19 |
| unmanipulated - low | 66 | 0.21 | 66 | 0.06 |
| Positive as neutral |  |  |  |  |
| high - unmanipulated | 66 | 0.04 | 66 | 0.09 |
| high - low | 66 | 0.18 | 66 | 0.33 |
| unmanipulated - low | 66 | 0.23 | 66 | 0.28 |
| Negative as positive |  |  |  |  |
| high - unmanipulated | 66 | 0.05 | 66 | 0.18 |
| high - low | 66 | 0.12 | 66 | 0.24 |
| unmanipulated - low | 66 | 0.18 | 66 | 0.10 |
| Negative as neutral |  |  | 66 |  |
| high - unmanipulated | 66 | 0.36 | 66 | 0.07 |
| high - low | 66 | 0.06 | 66 | 0.06 |
| unmanipulated - low | 66 | 0.30 | 66 | 0.15 |
| Neutral as positive |  |  |  |  |
| high - unmanipulated | 66 | 0.22 | 66 | 0.20 |
| high - low | 66 | 0.02 | 66 | 0.00 |
| unmanipulated - low | 66 | 0.22 | 66 | 0.20 |
| Neutral as negative |  |  |  |  |
| high - unmanipulated | 66 | 0.29 | 66 | 0.02 |
| high - low | 66 | 0.31 | 66 | 0.07 |
| unmanipulated - low | 66 | 0.05 | 66 | 0.08 |

| **Misclassifications** | **N** | ***d*** | **N** | ***d*** |
| --- | --- | --- | --- | --- |
|  | contrast | | saturation | |
| Positive as negative – positive as neutral | 66 | 0.74 | 66 | 0.15 |
| Positive as negative – negative as positive | 66 | 0.73 | 66 | 0.32 |
| Positive as negative – negative as neutral | 66 | 1.23 | 66 | 0.44 |
| Positive as negative – neutral as positive | 66 | 0.35 | 66 | 0.22 |
| Positive as negative – neutral as negative | 66 | 1.29 | 66 | 0.76 |
| Positive as neutral – negative as positive | 66 | 1.30 | 66 | 0.42 |
| Positive as neutral – negative as neutral | 66 | 0.90 | 66 | 0.41 |
| Positive as neutral – neutral as positive | 66 | 0.98 | 66 | 0.36 |
| Positive as neutral – neutral as negative | 66 | 0.26 | 66 | 0.49 |
| Negative as positive – negative as neutral | 66 | 1.90 | 66 | 0.69 |
| Negative as positive – neutral as positive | 66 | 0.54 | 66 | 0.15 |
| Negative as positive – neutral as negative | 66 | 1.52 | 66 | 0.77 |
| Negative as neutral – neutral as positive | 66 | 1.72 | 66 | 0.62 |
| Negative as neutral – neutral as negative | 66 | 0.39 | 66 | 0.15 |
| Neutral as positive – neutral as negative | 66 | 1.37 | 66 | 0.69 |

*Note*. d = effect size (Cohen’s d).

| **TABLE S8 Stimuli from the adapted stimulus set by Matzke and colleagues (2014) used for the FEST-1 (contrast)** | | |
| --- | --- | --- |
| **Stimulus Code** | | |
| 05F_FE_C_40 | 05F_HA_C_40 | 05F_NE_C_00 |
| 06F_FE_C_40 | 06F_HA_C_40 | 06F_NE_C_00 |
| 07F_FE_C_40 | 07F_HA_C_40 | 07F_NE_C_00 |
| 08F_FE_C_40 | 08F_HA_C_40 | 08F_NE_C_00 |
| 09F_FE_C_40 | 09F_HA_C_40 | 09F_NE_C_00 |
| 20M_FE_C_40 | 20M_HA_C_40 | 20M_NE_C_00 |
| 21M_FE_C_40 | 21M_HA_C_40 | 21M_NE_C_00 |
| 23M_FE_C_40 | 23M_HA_C_40 | 23M_NE_C_00 |
| 28M_FE_C_40 | 28M_HA_C_40 | 28M_NE_C_00 |
| 30M_FE_C_40 | 30M_HA_C_40 | 30M_NE_C_00 |

| **TABLE S9 Stimuli from the original NimStim Face Stimulus Set (Tottenham et al., 2009) used for the FEST-2 (saturation)** | | |
| --- | --- | --- |
| **Stimulus Code** | | |
| 05F_FE_C | 05F_HA_C | 05F_NE_C |
| 06F_FE_C | 06F_HA_C | 06F_NE_C |
| 07F_FE_C | 07F_HA_C | 07F_NE_C |
| 08F_FE_C | 08F_HA_C | 08F_NE_C |
| 09F_FE_C | 09F_HA_C | 09F_NE_C |
| 20M_FE_C | 20M_HA_C | 20M_NE_C |
| 21M_FE_C | 21M_HA_C | 21M_NE_C |
| 23M_FE_C | 23M_HA_C | 23M_NE_C |
| 28M_FE_C | 28M_HA_C | 28M_NE_C |
| 30M_FE_C | 30M_HA_C | 30M_NE_C |

**References**

Abler, B., and Kessler, H. (2009). Emotion regulation questionnaire–Eine deutschsprachige Fassung des ERQ von Gross und John. *Diagnostica* 55**,** 144-152.

Beierlein, C., Kemper, C., Kovaleva, A. J., & Rammstedt, B. (2014). "Interpersonales Vertrauen (KUSIV3).", in: *Zusammenstellung sozialwissenschaftlicher Items und Skalen (ZIS).*).

Erkic, M., Bailer, J., Fenske, S.C., Schmidt, S.N.L., Trojan, J., Schroder, A., Kirsch, P., and Mier, D. (2018). Impaired emotion processing and a reduction in trust in patients with somatic symptom disorder. *Clin Psychol Psychother* 25**,** 163-172.

Freitag, C.M., Retz-Junginger, P., Retz, W., Seitz, C., Palmason, H., Meyer, J., Rösler, M., and Von Gontard, A. (2007). Evaluation der deutschen version des Autismus-Spektrum-Quotienten (AQ)-die Kurzversion AQ-k. *Zeitschrift für klinische Psychologie und Psychotherapie* 36**,** 280-289.

Fydrich, T. (2002). "SPAI - Soziale Phobie und Angst Inventar.," in *Diagnostische Verfahren in der Psychotherapie,* ed. E. Brähler, Schumacher, J., Strauß, B. (Göttingen: Hogrefe), 335-338.

Huq, S.F., Garety, P.A., and Hemsley, D.R. (1988). Probabilistic judgements in deluded and non-deluded subjects. *Q J Exp Psychol A* 40**,** 801-812.

Klein, C., Andresen, B., and Jahn, T. (1997). Erfassung der schizotypen Persönlichkeit nach DSM-III-R: Psychometrische Eigenschaften einer autorisierten deutschsprachigen Übersetzung des "Schizotypal Personality Questionnaire" (SPQ) von Raine. [Psychometric assessment of the schizotypal personality according to DSM-III-R criteria: Psychometric properties of an authorized German translation of Raine's "Schizotypal Personality Questionnaire" (SPQ).]. *Diagnostica* 43**,** 347-369.

Kovaleva, A., Beierlein, C., Kemper, C., and Rammstedt, B. (2012). "Eine Kurzskala zur Messung von Impulsivität nach dem UPPS-Ansatz: Die Skala Impulsives-Verhalten-8 (I-8)". Gesis).

Lang, P., and Bradley, M.M. (2007). The International Affective Picture System (IAPS) in the study of emotion and attention. *Handbook of emotion elicitation and assessment* 29**,** 70-73.

Paulus, C. (2009). Der Saarbrücker Persönlichkeitsfragebogen SPF (IRI) zur messung von empathie: psychometrische evaluation der deutschen version des interpersonal reactivity index.

Schettino, A., Loeys, T., Bossi, M., and Pourtois, G. (2012). Valence-specific modulation in the accumulation of perceptual evidence prior to visual scene recognition. *PLoS One* 7**,** e38064.

Strobel, A., Beauducel, A., Debener, S., and Brocke, B. (2001). Eine deutschsprachige Version des BIS/BAS-Fragebogens von Carver und White. [A German version of Carver and White's BIS/BAS scales.]. *Zeitschrift für Differentielle und Diagnostische Psychologie* 22**,** 216-227.
